# Dry adhesives from carbon nanofibers grown in an open ethanol flame

**DOI:** 10.3762/bjnano.8.271

**Published:** 2017-12-15

**Authors:** Christian Lutz, Julia Syurik, C N Shyam Kumar, Christian Kübel, Michael Bruns, Hendrik Hölscher

**Affiliations:** 1 Institute of Microstructure Technology, Karlsruhe Institute of Technology (KIT), Hermann-von-Helmholtz-Platz 1, 76344 Eggenstein-Leopoldshafen, Germany; 2 Department of Materials and Earth Sciences, Technische Universität Darmstadt, 64287 Darmstadt, Germany; 3 Institute of Nanotechnology, Karlsruhe Institute of Technology (KIT), Hermann-von-Helmholtz-Platz 1, 76344 Eggenstein-Leopoldshafen, Germany; 4 Karlsruhe Nano Micro Facility (KNMF), Karlsruhe Institute of Technology (KIT), Hermann-von-Helmholtz-Platz 1, 76344 Eggenstein-Leopoldshafen, Germany; 5 Institute for Applied Materials, Karlsruhe Institute of Technology (KIT), Hermann-von-Helmholz-Platz 1, 76344 Eggenstein-Leopoldshafen, Germany

**Keywords:** adhesion, atomic force microscopy, carbon nanofibers

## Abstract

Based on magnetic-field-assisted growth of carbon nanofibers in an open ethanol flame we fabricated arrays of carbon nanofibers with different degrees of orientation. Inspired by the dry adhesive system of geckos we investigated the adhesive properties of such carbon nanofiber arrays with ordered and random orientation. AFM-based force spectroscopy revealed that adhesion force and energy rise linear with preload force. Carbon nanofibers oriented by a magnetic field show a 68% higher adhesion (0.66 N/cm^2^) than the randomly oriented fibers. Endurance tests revealed that the carbon nanofiber arrays withstand 50.000 attachment/detachment cycles without observable wear.

## Introduction

One-dimensional carbon nanostructures (1D-CNs), such as carbon nanofibers (CNFs) and carbon nanotubes (CNTs) consisting of cylindrical graphitic sheets, are very promising materials for nanotechnology [1]. They are well known for their outstanding properties that make them the material of choice for many applications [2]. In general, 1D-CNs grow via catalytic centers, typically transition metals such as iron, cobalt or nickel, in the constant presence of a carbon source at temperatures ranging from several hundred up to over thousand degrees Celsius in a closed chamber. The standard process for their growth is chemical vapor deposition (CVD) [3], which results in randomly oriented structures, whereas a plasma-enhanced CVD (PECVD) [4] allows for the growth of aligned structures. During growth of 1D-CNs, oxidized catalytic centers reduce into their pure state under hydrogen or ammonia treatment. These processes require a comparably complex infrastructure, a certain amount of process gases and huge energy input. There exist, however, alternative methods for CNT and CNF growth which are surprisingly simple [5–9]. They need only an open flame, which serves as the carbon source and provides the necessary temperature. Li and Hsieh demonstrated the growth of multiwalled carbon nanotubes (MW-CNTs) from the flame of a paraffin wax candle [7] and a Bunsen burner [8]. Pan and co-workers grew CNTs and CNFs with an ethanol flame [5] and demonstrated possible alignment during growth with an external electric [6] or magnetic field [9]. Surprisingly, these alternative growth methods for 1D-CNs did not receive much attention so far.

One among many promising applications of carbon nanotubes are dense arrays that feature interesting adhesion properties [10–18]. This utilization is inspired by geckos, which have very impressive adhesion properties, originating from thousands of hierarchically arranged hairs covering their toes. The smallest hairs with a tip diameter of about 200 nm efficiently get in contact with nearly every surface and adhere to it due to van der Waals forces, allowing the gecko to stick and climb nearly every surface [19–21]. Mimicking these nanostructures can lead to high performance dry adhesives with a great range of possible applications in attachment systems of climbing robots [22], manufacturing processes to transfer objects [23] and plasters in medicine [24]. Polymer-based dry adhesives [25–27] benefit from easy fabrication routes and low production cost. However, there are several polymer-related problems, such as thermal instability at elevated temperatures and creep [28]. Additionally, they are not applicable under conditions of high radiation like in outer space. Carbon nanotubes, however, benefit from excellent thermal stability up to 750 °C in air and 2800 °C in vacuum [29], alongside a high mechanical strength with a Young’s modulus of 0.8 TPa and a tensile strength of 150 GPa [30]. CNTs act similarly to the hairs of a Gecko, due to their diameters in the nanometer-range, they can bend quite easily when getting in contact with a rough surface. This effect enables effective contact splitting [31], which leads to an increased contact area, resulting in a high adhesion force. Furthermore, dry adhesives made from 1D-CNs do not suffer from creep, cosmic radiation, or vast temperature changes. Consequently, they are of great interest for applications under harsh conditions such as space technology. However, it is a challenge to grow CNTs or CNFs with uniform morphology on large areas in a cost-effective way.

Here, we examine the CNF growth process based on an open ethanol flame with the option to apply a magnetic field. With this method we fabricated randomly oriented and oriented CNFs and investigated their adhesion properties by atomic force microscopy (AFM). Both types of CNF arrays withstand long-term endurance tests. The oriented CNFs feature higher adhesion as the non-oriented ones.

## Experimental

**Figure 1:**
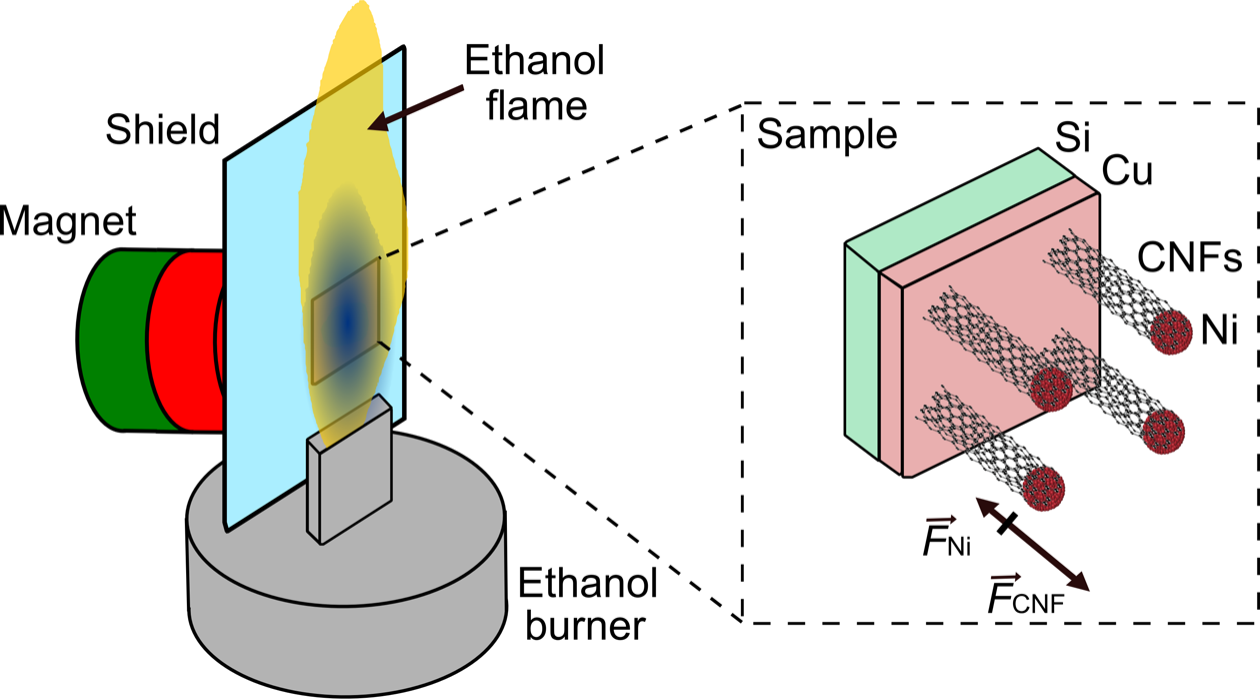
Schematic setup for the growth of carbon nanofibers in an open ethanol flame. The sample is placed in the center of the flame. A silicon shield protects the permanent magnet from the heat of the flame when the optional magnetic field is applied. The filed helps to orient the CNFs in a predominant direction during their growth. The sample consists of a piece of a silicon wafer with a 60 nm thick copper layer covered with nickel catalysts from which the CNFs grow. During the growth with the magnet a diamagnetic force acts on the CNFs (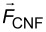) in the direction away from the magnet. Additionally, depending on the actual temperature a ferromagnetic or paramagnetic force acts on the Ni catalysts. Since the temperature of 750 °C in the ethanol flame is considerably higher than the Curie temperature of Ni (360 °C) only a small paramagnetic force acts on the Ni catalysts (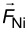) in the direction towards the magnet. Consequently, as the paramagnetic force is smaller than the diamagnetic force, the net force acts away from the magnet orienting the growing CNFs.

For our experimental setup we utilize a standard ethanol burner with a 2 mm × 12 mm wick (Figure 1) and a combustion rate of 0.4 mL/min. The ethanol flame heats the sample and serves as carbon source at the same time. A 10 × 10 mm^2^ large piece of a silicon wafer covered with an evaporated 60 nm thick copper layer serves as substrate. A 2 μL droplet of a NiCl_2_·6H_2_O (Sigma-Aldrich) solution in ethanol with a concentration of 20 mg/mL is casted on the sample and dried in air before subsequent processing. After that, the substrate is placed directly in the ethanol flame at a height of 2 mm over the wick. Measurement with a thermocouple confirmed that the temperature in the center of the sample reaches 750 °C. Typical growth times are ca. 3 min.

Optionally, a permanent magnet with a calculated magnetic flux of 506 mT at the magnet surface was built-in by stacking five cylindrical neodymium magnets (Maqna, Otom Group GmbH, Grade 45, 3 mm thick, 25 mm in diameter). The distance between magnet and sample was 3 mm yielding a magnetic flux of about 387 mT at the surface of the sample. To prevent the magnets from losing their magnetization because of the elevated temperatures, we placed a piece of a silicon wafer between flame and magnet acting as heat shield.

The overall morphology of the grown carbon nanostructures was investigated by scanning electron microscopy (SEM, Zeiss SUPRA 60 VP) and high-resolution transmission electron microscopy (HRTEM, FEI Titan 80-300). TEM measurements were performed at 80 kV operation voltage and images acquired using a Gatan US1000 CCD camera. TEM samples were prepared by scraping the grown carbon nanostructures from the substrate directly on carbon-coated copper grids (Quantifoil). Raman spectroscopy was performed with an excitation wavelength of 532 nm (Renishaw inVia Raman microscope).

X-ray photoelectron spectroscopy (XPS) measurements were performed using a K-Alpha+ XPS instrument (Thermo Fisher Scientific, East Grinstead, UK). Data acquisition and processing using the Thermo Avantage software is described elsewhere [32]. All samples were analyzed using a micro-focused, monochromated Al Kα X-ray source (30–400 μm spot size). The K-Alpha charge compensation system was employed during analysis, using electrons of 8 eV energy and low-energy argon ions to prevent any localized charge build-up. The spectra were fitted with one or more Voigt profiles (binding energy uncertainty: ±0.1 eV). All spectra were referenced to the C 1s peak of hydrocarbons at 285.0 eV binding energy, controlled by means of the well-known photoelectron peaks of metallic Cu, Ag, and Au. Sample cleaning to remove organic contaminations was performed with the Thermo Scientific MAGCIS (Mono Atomic and Gas Cluster Ion Source) using Ar_1000_+ clusters at 8 keV primary energy and a raster size of 2 × 4 mm^2^.

The adhesion force and energy were determined from force–distance curves measured with an AFM (Dimension Icon, Bruker). In order to have a defined contact, a 20 μm SiO_2_ sphere was glued to the end of a tipless silicon cantilever (All In One-TL from BudgetSensors) using the approach of Mak and co-workers [33]. We confirmed by SEM that no glue was left on the top of the SiO_2_ sphere (see the insert in Figure 7 below). For the adhesion measurements a constant ramp rate of 1.5 μm/s was applied (adhesion measurement with ramp rates between 0.2 and 8 μm/s showed similar results). The spring constant of the cantilever was determined to 7.74 N/m with the thermal tune method [34] integrated in the AFM software. All the measurements presented here were conducted with the same cantilever.

## Results and Discussion

### Growth of carbon nanofibers

Inspired by the study of Zhang and Pan [9], we aligned the sample, the magnet and the shield parallel to the ethanol flame with a 90° tilt (see Figure 1). The advantage of this setup is that the magnet heats up only slowly, because the hot air from the ethanol flame rises upwards. At the end of a three-minute experiment, the temperature of the magnet is only about 40 °C, which is well in the stable operation range of the utilized type of magnet (*<*80 °C). In contrast to previous studies, we used NiCl_2_·6H_2_O as catalyst with a 60 nm thick copper layer on a silicon wafer. Interestingly, this allows us to run the growth procedure without the usual reduction step in which hydrogen reduces the nickel catalyst into its pure state before growth [35]. Based on our observations, we conclude that the 60 nm copper layer on the Si substrate plays an important role for the reduction of the nickel catalysts because experiments with nickel on pure Si or SiO_2_ substrates without copper show no or diminishing CNF growth. Kumar et al. [36] reported that copper–nickel catalysts are very efficient to produce hydrogen in an ethanol flame. This means that nickel is reduced to a pure state by hydrogen created in the ethanol flame.

**Figure 2:**
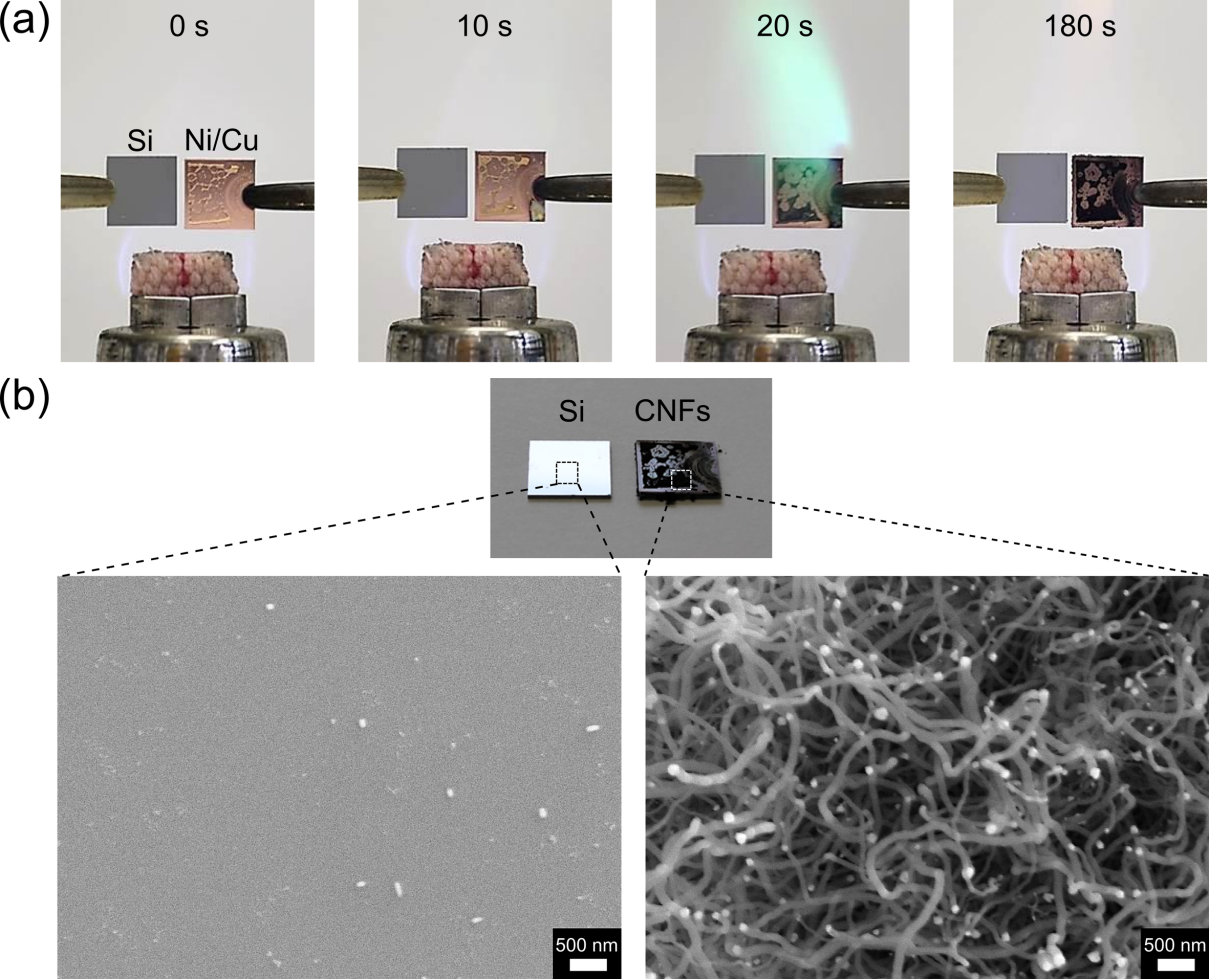
(a) Time series of an experiment showing the growth of CNFs in an open ethanol flame. For comparison we show a clean silicon sample as reference on the left and a copper substrate with Ni-containing catalysts on the right side. (b) SEM images of the samples after this 3 minute experiment show that CNFs grow only on the copper substrate with Ni-containing catalysts. No CNFs were observed on the reference sample.

Our vertical setup results in a fairly stable ethanol flame in the sample area due to a constant flow of the ethanol flame compared to the case of a horizontal placement of the sample as suggested by Zhang and Pan [9]. Figure 2a shows a time series of photos taken during a typical experiment during which CNFs are grown in an open ethanol flame. The right sample is a copper substrate with Ni-containing salt. The left sample is a clean silicon substrate without catalyst. After 20 s, the ethanol flame went green suggesting that NiCl_2_·6H_2_O is transformed to Ni-containing catalysts. Shortly after that, the area initially covered with Ni-containing salt, became black indicating the growth of carbon structures. The SEM images of samples taken after such a three-minute experiment (Figure 2b) indicate that carbon nanostructures grow only on the copper substrate with Ni-containing catalysts while no structures were observed on the clean silicon sample. The samples with carbon nanostructures are patchy. Areas covered with 1D-CNs are limited to spots of the ethanol flame with nearly constant process conditions and a temperature in the range of 750 °C. Another limitation is the process stability, especially the environmental conditions. Humidity and temperature are critical factors for the successful growth of CNFs in an open flame as discussed below.

**Figure 3:**
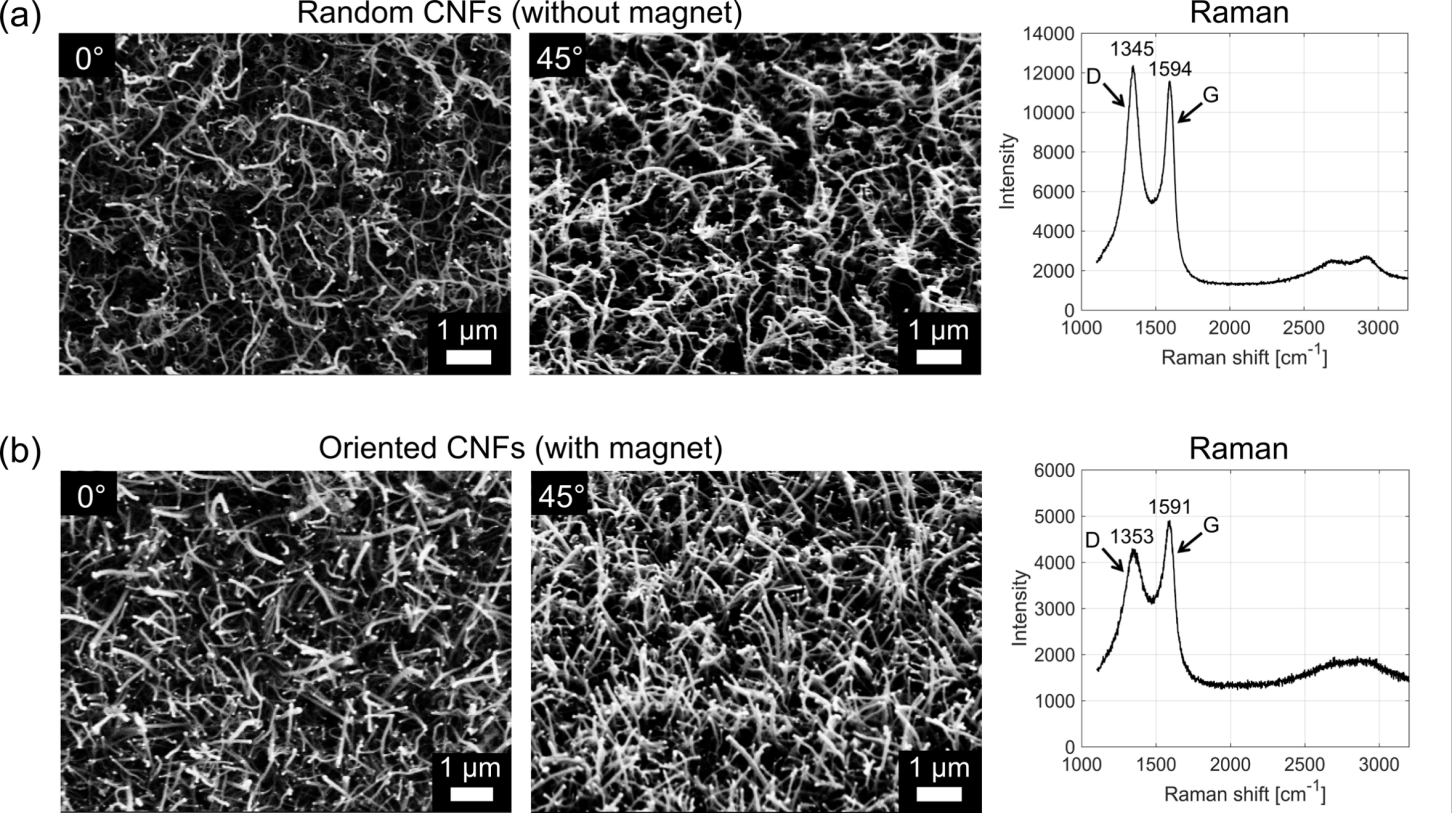
Morphology and Raman spectra of the obtained CNFs for (a) randomly oriented CNFs and (b) oriented CNFs. The SEM images show the CNF arrays under different angles. Raman measurements, conducted with a laser excitation of 532 nm, show the D and G bands characteristic for carbon materials.

Randomly oriented CNFs were observed when no magnetic field was applied (Figure 3a). The catalytic particles can be seen at the end of each nanofiber, indicating a tip-growth mechanism of the CNFs. The bright points in the SEM images most likely correspond to nickel-containing catalysts, which are still present after growth. Experiments with Si, SiO_2_ and Cu without Ni-containing salt show no CNF growth. As Ni is the only material that can act as a catalyst to grow CNFs in our process, we conclude that the material observed at the end of the CNFs is Ni-based, as suggested by Chai and co-workers [37]. However, it might be possible that the catalytic particles are an alloy of Ni and small amounts of Cu. An improvement of the CNFs orientation is observed with the use of the magnet (Figure 3b). In this case CNFs are mostly orientated away from the magnetic field, leading to a different morphology and adhesion as discussed below. This effect is most likely caused by the diamagnetism of the CNFs [38] despite of the paramagnetism of the Ni catalysts. During the growth in the ethanol flame, the magnetic field causes a diamagnetic force onto the CNFs (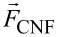) acting away from the magnet. In principle there is a ferromagnetic force acting on the Ni catalysts below the Curie temperature of Nickel (360 °C). The temperature of the ethanol flame, however, is about 750 °C. Consequently, only a small paramagnetic force might act on the Ni catalysts (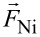) and the direction of the resulting force orients the CNFs away from the magnet as sketched in Figure 1.

The diameters of the randomly oriented and the oriented CNFs are between 40 and 80 nm with a length of about 3 μm as determined by SEM. The height of the oriented CNFs, measured from the base to the tip of the CNFs, is ca. 3 μm, while the height of the randomly oriented CNFs is ca. 2 μm. The growth rate was about 1 μm/min. Raman measurements show D and G bands for both structures, which are characteristic for carbon materials [39]. The D-band is caused by a disordered structure in CNFs and other carbon materials, indicating defects in sp^2^-hybridized carbon. The G-band indicates stretching of the C–C bonds, characteristic for CNTs, CNFs, or other graphitic materials [40]. The D/G ratio is a measure of disorder in nanofibers. The randomly oriented CNFs show a significantly higher value than the oriented CNFs (0.87 for oriented CNFs and 1.06 for randomly oriented CNFs), suggesting a higher graphitic degree of ordering of the oriented CNFs.

**Figure 4:**
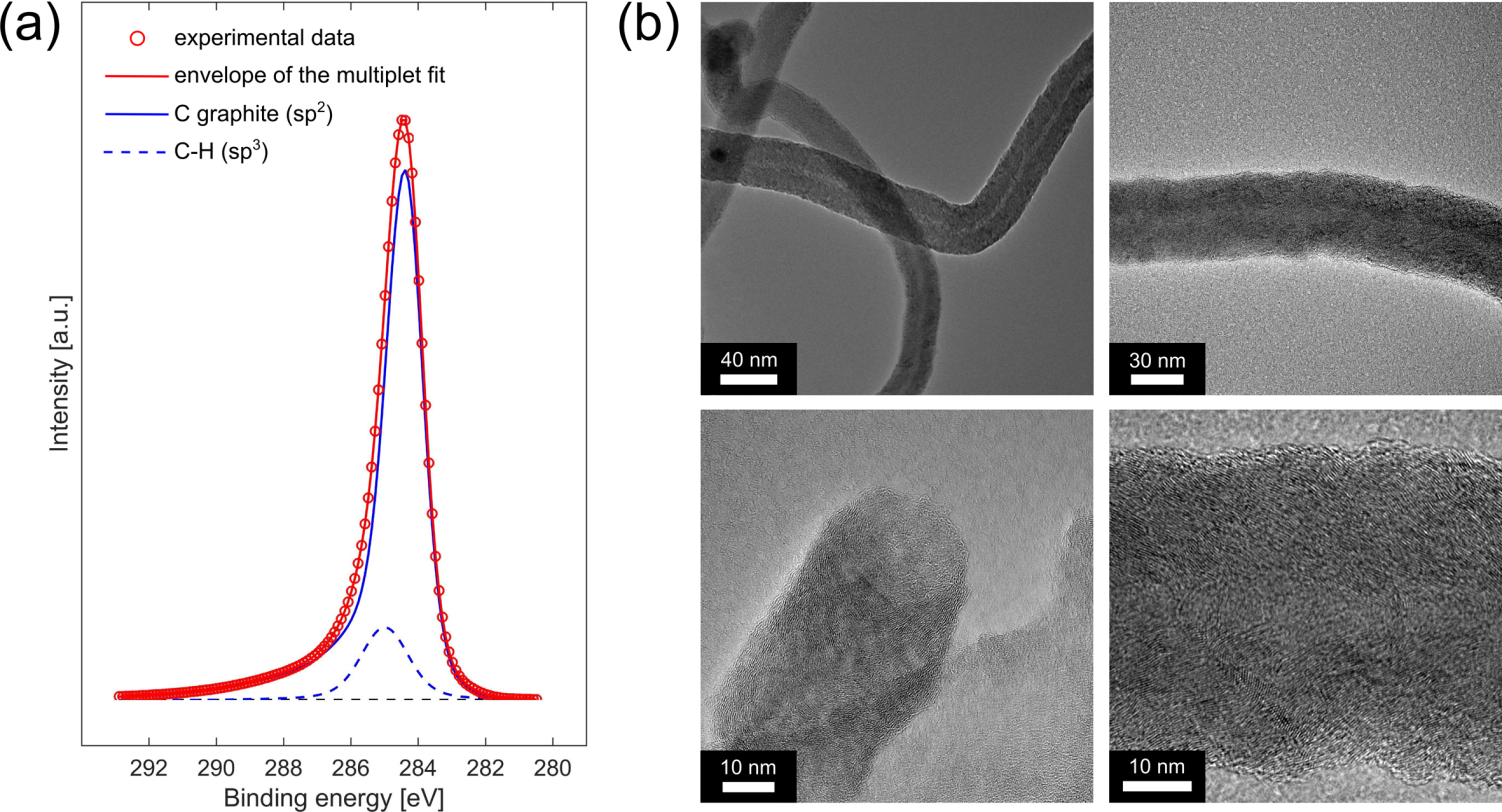
(a) C 1s XP spectrum of the grown carbon structures. The main component at 284.4 eV indicates sp^2^-hybridized carbon (blue solid line) and the weak component at 285.0 eV is stemming from adventitious sp^3^-hybridized carbon (blue dashed line). (b) HRTEM images of the grown CNFs.

We conducted XPS experiments of some typical samples. The main C 1s peak at 284.4 eV in Figure 4 a doubtlessly proves graphitic carbon sp^2^ (blue solid line) and is in a good agreement with XPS investigations of CNFs by other authors [41–42]. The weak component at 285.0 eV (blue dashed line) originates from so-called ’adventitious carbon’ sp^3^, describing hydrocarbon contamination due to the exposure to ambient atmosphere. The HRTEM images in Figure 4 b reveal somewhat disordered bamboo or herringbone like CNFs. Additionally, some structures show a hollow interior. The overall outcome of our SEM, Raman, XPS and TEM experiments confirms that the nanostructures we observe on our samples are 1D-CNs with mostly CNFs and a lower amount of CNTs.

**Figure 5:**
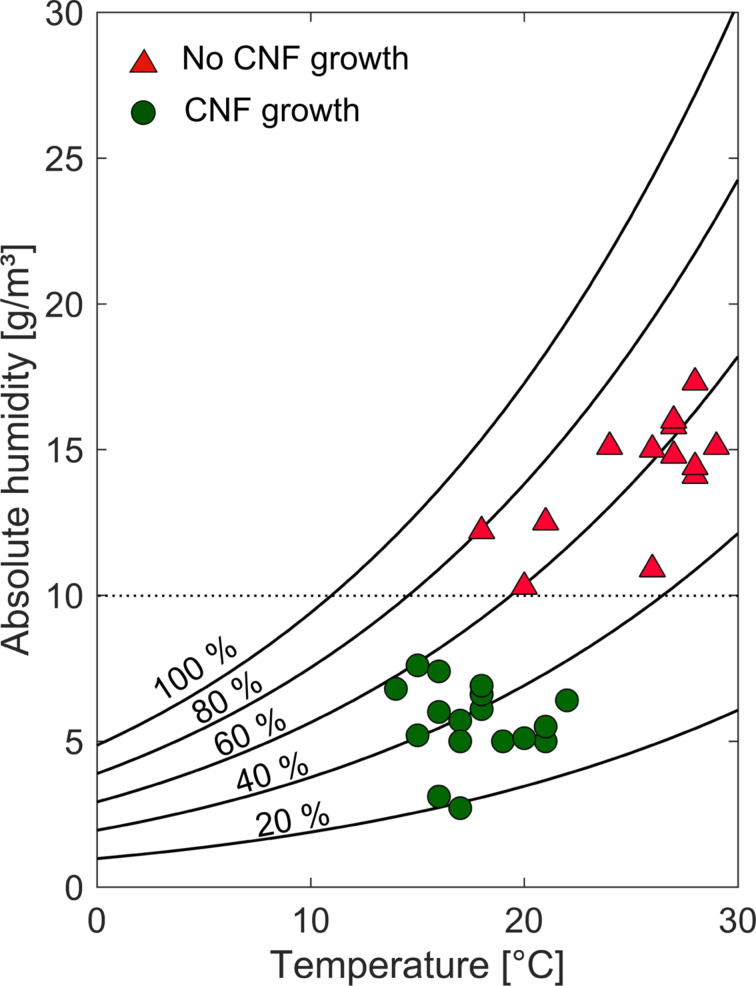
Summary of experiments resulting in CNF growth (green circles) or in no CNF growth (red triangles). All experiments are compiled in a diagram of absolute humidity as a function of the temperature. The thin solid lines represent the relative humidity. In general, CNFs grow at lower temperature and humidity. All experiments conducted with an absolute humidity larger than about 10 g/m^3^ were not successful.

Although we regularly find such 1D-CNs of different qualities on our samples, it should be mentioned that the quality of the resulting arrays obtained by the magnetic-field assisted growth in an ethanol flame, depends on the environmental conditions, i.e., temperature and humidity. Summarizing about 30 experiments conducted all over the year under different environmental conditions, we observed that a growth of CNFs is, in general, only successful at lower ambient lab temperatures and humidity as shown in Figure 5. We, therefore, assume that water condenses on the hygroscopic NiCl_2_·6H_2_O catalytic layer at elevated temperatures (above ca. 25 °C) and humidity values (above ca. 50%). The critical relative humidity of this salt is about 54% at 20 °C [43]. Therefore, condensed water might oxidize the bottom copper layer in this humidity and temperature range. Such an oxide layer might, subsequently, prevent CNF growth. Another important factor for successful CNF growth is the stability of the ethanol flame. Flicker of the flame, causing unpredictable short-term rapid temperature drops, might lead to re-oxidation of previously reduced catalysts and might hinder continuous CNF growth.

### Adhesion properties

Inspired by previous studies [10–18], we conducted adhesion measurements of the obtained oriented and randomly oriented CNFs. Since the area covered with CNFs is limited on our samples we choose atomic force microscopy for these measurements. As AFM enables a very precise measurement in the nanonewton-range on small areas, it is in our opinion perfect to investigate the adhesion of CNF samples.

**Figure 6:**
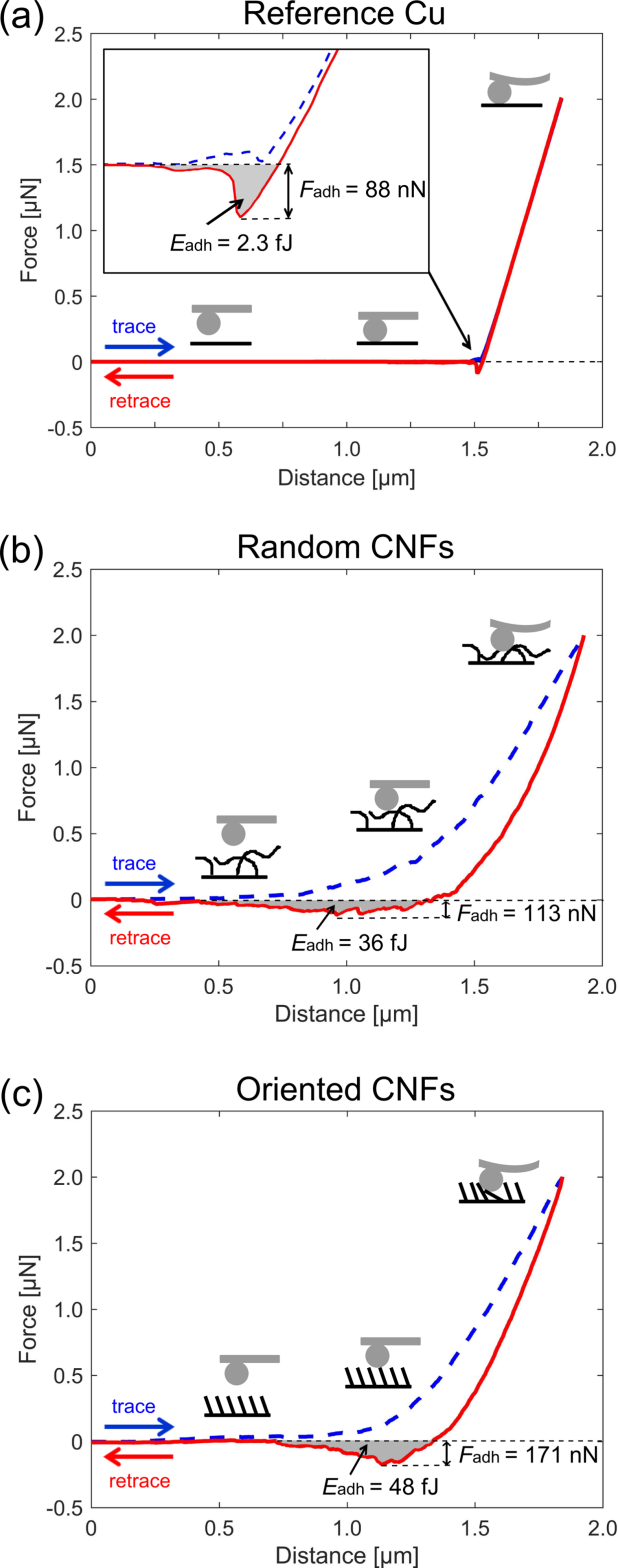
Force–distance diagrams obtained through atomic force microscopy. A spherical tip with a diameter of 20 μm was brought in contact to the surface with a preload force of 2 μN and pulled off. During this process, the force–distance diagram is recorded for (a) the reference copper surface, (b) randomly oriented CNFs and (c) oriented CNFs. The lines in the plots are trace (blue dashed line) and retrace (red solid line). The adhesion force (*F*_adh_), defined as the force required to separate (lift off) the cantilever from the surface is indicated in the diagram. The adhesion energy (*E*_adh_), is the shaded area between zero line and retrace force curve. The oriented CNFs show higher adhesion force and energy, compared to the randomly oriented CNFs and the flat reference surface.

We prepared a flat reference sample. A copper substrate was processed in the ethanol flame but without catalysts and, hence, no CNF growth. The adhesion of this flat copper sample is compared with the adhesion of randomly oriented CNFs and oriented CNFs in Figure 6. The two curves in the diagrams represent trace (dashed blue line) and retrace (solid red line). The preload force was always set to 2 μN. The adhesion force is defined as the force that is necessary to lift the sphere glued to the AFM cantilever completely from the surface. This quantity is indicated as the lowest (negative) force in the diagrams. The adhesion energy is defined as the area between retrace and zero line. It corresponds to the energy necessary to free the sphere from the surface. The force–distance diagrams show considerably higher adhesion forces and energies for the CNF structures compared to the flat reference.

**Figure 7:**
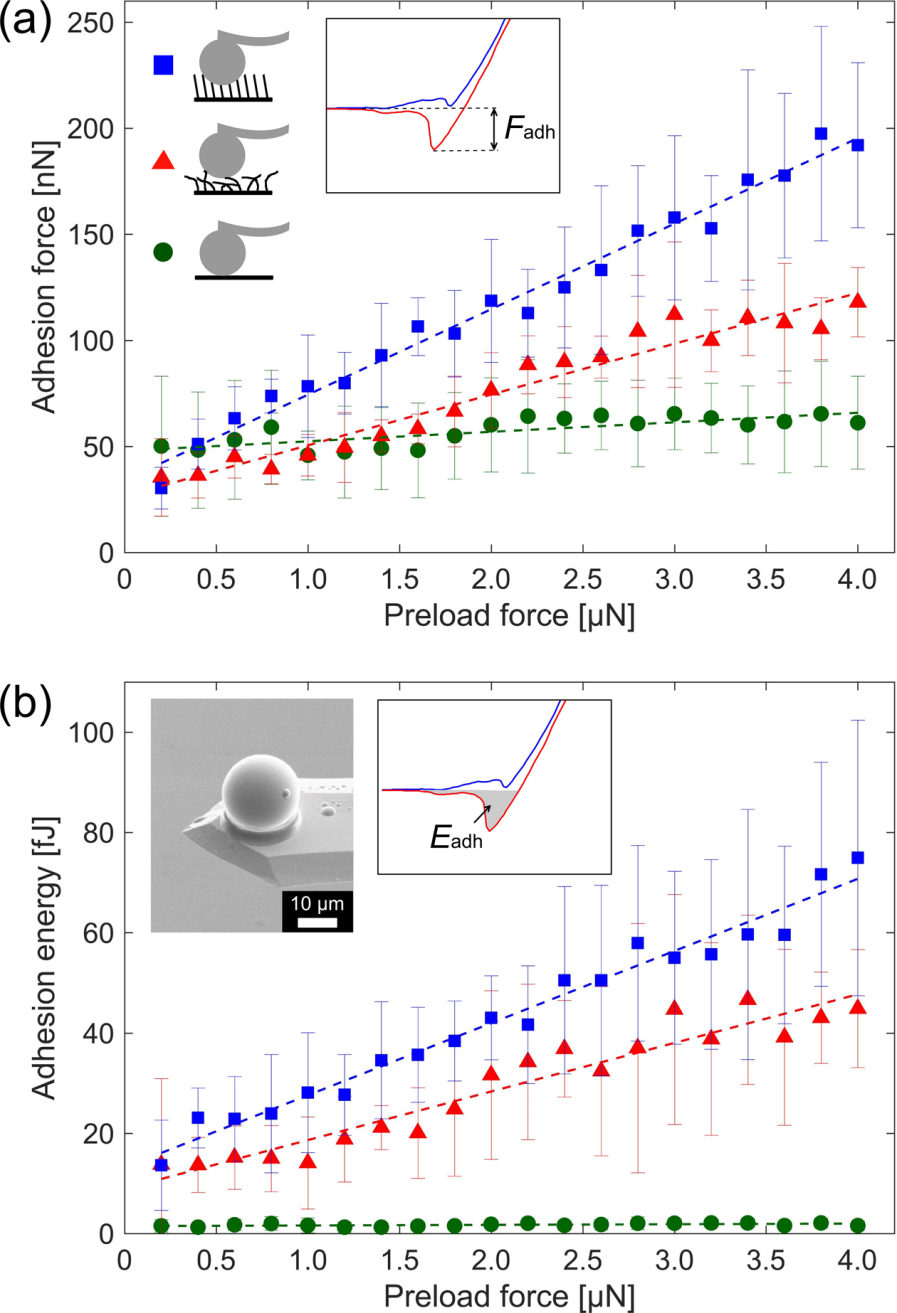
(a) The measured adhesion force as a function of the preload force and (b) the calculated adhesion energy as a function of the preload force. The symbols correspond to the oriented CNFs (blue squares), the randomly oriented CNFs (red triangles) and the flat reference (green circles). The dashed lines represent linear fits. The insert in panel (b) shows a SEM image of the AFM cantilever with the glued SiO_2_ sphere to conduct the adhesion measurements.

A set of adhesion measurements was conducted at six different positions of each sample with preload forces ranging from 0.2 to 4.0 μN. For all measurements we evaluated the mean value of adhesion force and energy. Figure 7a shows the adhesion forces and Figure 7b the calculated adhesion energies for oriented CNFs (blue squares), randomly oriented CNFs (red triangles) and the flat reference (green circles). The error bars represent the standard deviation from six measurements. The scatter of experimental values can be mainly explained by differences of the sample quality at the six analyzed positions, such as, different orientation degree or density of CNFs. Independent on the applied preload, the adhesion force of the oriented CNFs is higher, than that of randomly oriented CNFs. For small preload forces up to 0.6 μN, the adhesion of the plain copper substrate is comparable or higher than that of CNFs. This might be due to smaller contact area between CNFs and the silica sphere caused by a low preload force. The adhesion forces for oriented and randomly oriented CNFs, however, increase linearly with preload force whereas the adhesion force of the reference is nearly constant between 50 and 60 nN. The increase in adhesion is in agreement with the study of Ge and co-workers [44]. The reason for this is that a higher preload force brings more CNFs in contact with the silica sphere, which increases the adhesion force. The dashed lines represent the linear fits for oriented CNFs (*F*_adh_ = 0.040*F*_pre_ + 34 nN), randomly oriented CNFs (*F*_adh_ = 0.024*F*_pre_ + 27 nN) and the reference (*F*_adh_ = 0.004*F*_pre_ + 48 nN). Where *F*_adh_ is the adhesion force in nanonewtons and *F*_pre_ is the preload force in nanonewtons. The calculated adhesion energies for oriented and randomly oriented rise linearly with preload force, whereas the adhesion energies of the reference are nearly constant between 1 and 2 fJ. For the adhesion energies linear fits for oriented CNFs (*E*_adh_ = 0.014(fJ/nN)*F*_pre_ + 13 fJ), randomly oriented CNFs (*E*_adh_ = 0.007(fJ/nN)*F*_pre_ + 9 fJ) and the reference (*E*_adh_ = 0.0001(fJ/nN)*F*_pre_+ 1.5 fJ) were determined. Where *E*_adh_ is the adhesion energy in fJ and *F*_pre_ is the preload force in nanonewtons.

The adhesion force of oriented CNFs is 68% higher than that of the randomly oriented CNFs. Oriented CNFs show a maximum adhesion energy of 75 fJ at a preload force of 4 μN. Randomly oriented CNFs show a maximum adhesion energy of 47 fJ at a preload force of 3.4 μN. This can be explained with the contact-splitting theory [31] stating that adhesion rises with the number of contacts per area. From SEM images we estimated the number CNF apexes per area that can get in contact with the sphere. In the case of oriented CNFs, this density is in the range of 8 CNFs/μm^2^, while it is in the range of 5 CNFs/μm^2^ for randomly oriented CNFs. The reason for this is that randomly oriented CNFs are entangled, which prevents that some CNFs get in contact with the silica sphere, as schematically shown in Figure 7. Consequently, the oriented CNFs have higher adhesion forces and energies than the randomly oriented CNFs.

**Figure 8:**
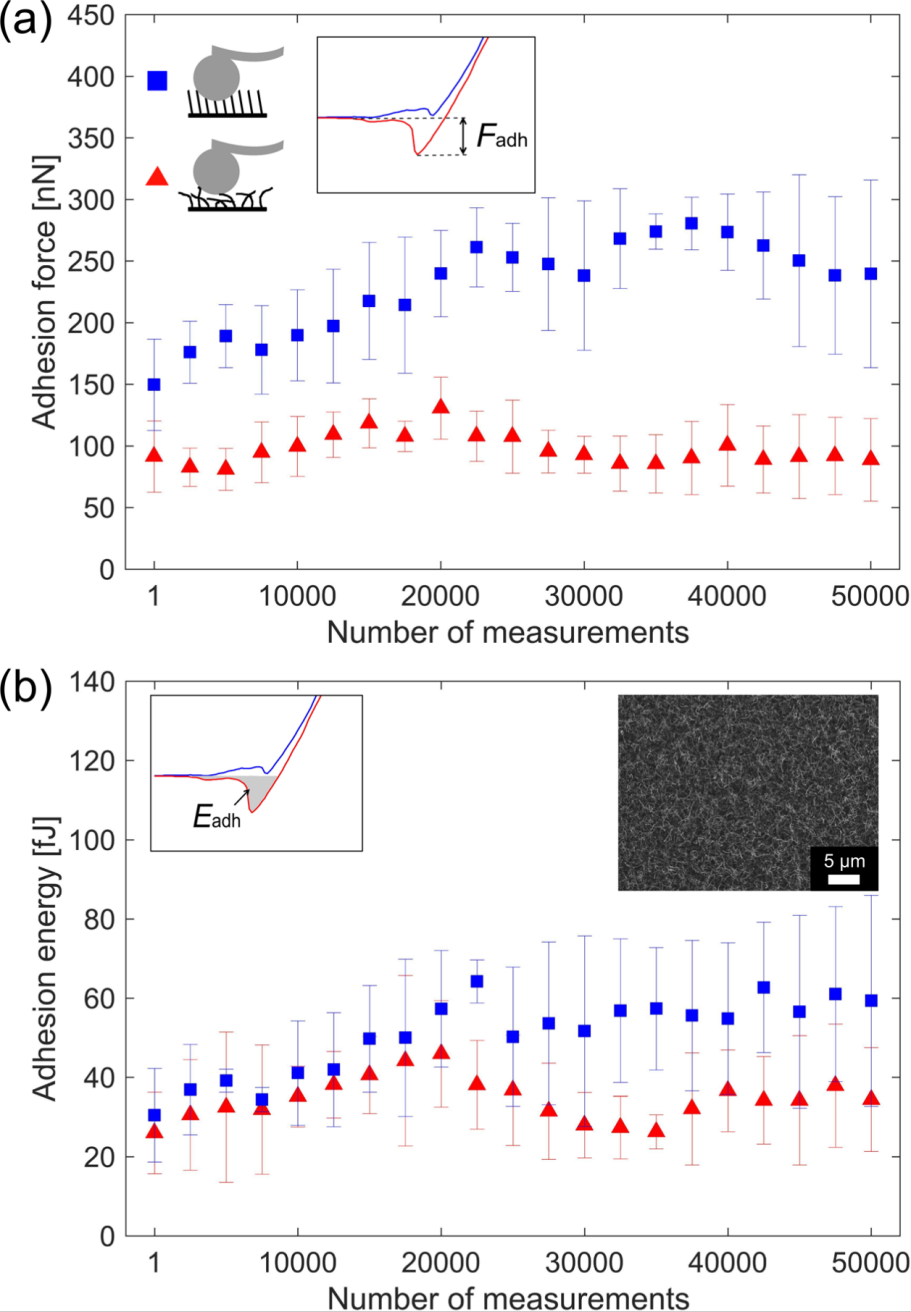
AFM-based long-term adhesion measurement with (a) the adhesion force and (b) the adhesion energy plotted as a function of the number of measurements. The measurements for the oriented CNFs (blue squares) and the randomly oriented CNFs (red triangles) show that the CNF-based structures under test are stable up to 50000 measurements. This indicates their potential for reusable dry adhesives. The insert in panel (b) shows an SEM image of an array with CNFs after 50000 approach-and-retraction cycles.

An important question for dry adhesives is their long-term stability. It is essential for various applications, for instance, for the number of steps a climbing robot can execute before the adhesive layer needs to be replaced. Endurance tests, however, are time-consuming, which is the reason why most studies of CNF adhesives limit their adhesion measurement to a few cycles [11]. Here, we analyze the endurance of the obtained CNF arrays, with a set of three long-term endurance tests with 50000 approach-and-retraction cycles. The preload force of 2 μN was kept constant during all measurements. The error bars are the standard deviation of three endurance runs. The values of adhesion force and energy of randomly oriented CNFs and oriented CNFs are nearly constant for all 50000 measurements (Figure 8). SEM investigations of the CNF arrays after 50000 approach-and-retraction cycles showed no visible damage (see insert in Figure 8b). The maximum recorded adhesion force was 280 nN for the oriented CNFs. Calculated from the touching area of the spherical cantilever (projected area), which is approximately 42 μm^2^, the adhesion strength corresponds to 0.66 N/cm^2^. This is considerably lower as the adhesion of real gecko footpads (10 N/cm^2^) [19], but the adhesion strength might be improved by smaller CNF diameters leading to a higher density and improvement of the CNF orientation resulting in more CNFs in contact with the surface. Additionally, hierarchically structured CNFs, such as Y-shaped CNFs [45–46], might help to better mimic a real gecko footpad, leading to an improved adhesion strength of artificial structures based on CNFs. Overall, the measurements demonstrate that CNF arrays have the potential for applications relying on adhesives with long-term stability under harsh environments.

## Conclusion

We presented a fabrication method to produce dry adhesives by the growth of CNFs in an open ethanol flame. The CNFs are randomly oriented. Alternatively, they can be oriented by applying a magnetic field during growth. The overall process has the advantage of not requiring a complex apparatus and process gases. The adhesion properties of the produced CNF arrays were analyzed by AFM. We confirmed that oriented CNFs have 68% higher adhesion force and energy than randomly oriented CNFs. Additionally, AFM endurance tests demonstrate that the CNF-based adhesives remain intact after up to 50000 cycles demonstrating their potential for long-term applications.

The introduced process to grow CNF-based dry adhesives is comparably simple and environmentally friendly. Only 1.2 mL ethanol are needed to produce one sample of roughly one square centimeter covered with CNFs with a growing time of 3 min. This is much less than the amount of process gases necessary to grow CNFs with conventional CVD methods. At the same time it avoids the use of unfavourable gases such as hydrogen or ammonia and electrical power for heating as the ethanol flame provides sufficiently high temperatures by itself. It is, of course, unlikely to produce CNFs on an industrial scale with an open flame under ambient conditions. It might be, however, a good starting point for the fabrication of CNFs on larger scales with the same chemical process but under more defined conditions. Finally, we think that the presented approach is especially useful for educational purposes, because it does not need an elaborate set-up.
